# Iridescence, polarisation and directionality of *Morpho* butterfly displays

**DOI:** 10.1007/s00359-026-01817-1

**Published:** 2026-06-04

**Authors:** Juliana Sosa Espinosa, Marco A. Giraldo, Doekele G. Stavenga, Casper J. van der Kooi

**Affiliations:** 1 Groningen Institute for Evolutionary Life Sciences, University of Groningen, Groningen, The Netherlands; 2 Biophysics Group, Institute of Physics, University of Antioquia, Medellin, Colombia

**Keywords:** Structural colouration, Gloss, Directionality, Spectrophotometry, Scatterometry

## Abstract

*Morpho* butterflies are widely known for their striking blue colourations, which are produced by multilayered ridges of the wing scales. Several *Morpho* species have a flashing iridescence, but others have a more diffuse display owing to curved wing scales. The structural colouration is even absent in species with whitish or brown colours, which suggests that there is a considerable intrageneric variation in wing scale anatomy and pigmentation. Here, we investigate four species with superficially similar blue wings: *Morpho aega*, *M. zephyritis*,* M. godartii* and *M. helenor*. Characterising the spectral and spatial reflection properties, we reveal marked interspecific differences in spectral and angle-dependent wing reflectance. We show that the superposition of scales broadens the angular range of the display, whereas the orientation of the scale ridges determines the directionality and polarisation of the reflected light. We discuss how the polarised iridescent displays of *Morpho* butterflies are presumably tuned to their natural environmental conditions and to the visual perception of their observers.

**Supplementary Information:**

The online version contains supplementary material available at 10.1007/s00359-026-01817-1.

## Introduction

*Morpho* butterflies display some of the most vibrant blue iridescent colourations found in nature. The genus *Morpho* contains 30 species that showcase remarkable variations in colour, wing size and shape. In some species, the colours are white or brownish, distinctly deviating from the striking blues (DeVries et al. 2010; Penz et al. 2012; Chazot et al. 2016, 2021; Giraldo et al. 2016). The blue colourations within the genus are structurally produced, being the result of the interaction of light with nanostructured wing scales (Srinivasarao 1999).

The wing scales are usually arranged at two imbricate layers, with cover scales overlapping the ground scales. The small (~ 100–200 μm) wing scales consist of two cuticle laminae connected by trabeculae. The adwing (or lower) lamina of the scale faces the wing substrate. It is a thin plate, which acts as an optical thin film reflector (Ghiradella 1998; Kinoshita and Yoshioka 2005; Giraldo and Stavenga 2016). The abwing (or upper) lamina consists of parallel ridges, connected by crossribs. The ridges are structures that run parallelly along the major axis of the scales and, in *Morpho*, are modified into stacks of lamellae, the number of which can be up to ten, depending on the species (Giraldo et al. 2016). The stacks of lamellae act as a multilayered reflector, creating a reflectance band in the blue wavelength range. In anatomical sections, the multilayers appear as a ‘Christmas tree’ (Vukusic and Sambles 2003; Kinoshita and Yoshioka 2005; Berthier et al. 2006; Berthier 2010; Giraldo and Stavenga 2016; Giraldo et al. 2016).

The ridges together act as a diffraction grating. Directional illumination of the wing scales produces a spatially restricted band-like reflection that is perpendicular to the direction of the ridges (Yoshioka and Kinoshita 2004, 2006; Giraldo et al. 2008; Stavenga et al. 2009; Giraldo and Stavenga 2016; Sosa Espinosa et al. 2024). As a result, the displays of some *Morpho* species are highly directional. In addition, these displays are also iridescent, as their colour changes upon varying angles of view or illumination (Srinivasarao 1999; Stuart-Fox et al. 2021). The most flashing, iridescent visual effects result from wings with flat scales (Sosa Espinosa et al. 2024). In contrast, disorder in the optical nanostructures results in both high reflectivity and a wider angular spread of light, thereby enhancing visual discriminability (Kinoshita and Yoshioka 2005; Dietz et al. 2025). The morphological traits that constrain the directionality of the visual displays in butterflies have been studied in only a few *Morpho* cases (Vukusic et al. 2001; Kinoshita et al. 2002; Berthier et al. 2007; Thomé et al. 2020; Sosa Espinosa et al. 2024).

The multilayer optics of *Morpho* butterflies have been studied in considerable detail, but we know less about how the morphology and stacking of wing scales determine the directionality of the visual display (Vukusic et al. 1999; Giraldo et al. 2016; Sosa Espinosa et al. 2024). As *Morpho* butterflies are a model to study the functional significance of iridescence in the evolution of flight behaviour, visual communication signalling and evasive mimicry (DeVries et al. 2010; Llaurens et al. 2021; Le Roy et al. 2021), it is crucial to better understand the spatial modulations of these displays. This includes polarization effects, referring to the way the orientation of light waves changes after being reflected by the butterfly wing (Berthier et al. 2006; Yoshioka and Kinoshita 2007). In this study, we examine four *Morpho* species that noticeably differ in flashing iridescence, degree of scale overlap and scale curvature, to investigate their visual display both spatially and spectrally, and their polarisation signature.

## Methods

### Specimens and (micro)photographs

We purchased two male individuals of *Morpho aega*, *M. zephyritis*, *M. godartii*, and *M. helenor* from commercial suppliers. In addition, for all species, we obtained intact wings of two individuals from the Muséum National d’Histoire Naturelle, Paris. Prior to experimentation, both preserved individuals and intact wings were cautiously examined to verify that scale alignment remained unaltered due to handling. The butterflies’ dorsal wing areas that display the dominant colouration were photographed using a Nikon D70 Camera equipped with an HD-130 macro-LED ring flash. To capture the polarised iridescent display, specimens were positioned horizontally and illuminated at an incident angle of ∼60° (Supplementary Material, Fig. S0C). The reflected light passed a linear polarising filter in front of the camera that was positioned at the mirroring angle. To visualize the scale arrangement on the wings, we performed brightfield epi-illumination microscopy, using a Zeiss Universal Microscope (Zeiss, Oberkochen, Germany) with a Zeiss Epiplan objective (8x/0.2).

### Spectrophotometry

Reflectance spectra of intact wings were measured using an integrating sphere (Avasphere-50, Avantes, Apeldoorn, the Netherlands) illuminated by a Deuterium-Halogen lamp (AvaLight D(H)-S), with a white reflectance standard (WS-2, Avantes) as a reference. In addition, angle-dependent reflectance spectra of 3 mm intact wing patches were measured using a goniometric setup consisting of two rotatable, coplanar optical fibres aligned in the plane that was parallel to the scale ridges (Supplementary Material, Fig. S0D). One fibre delivered light from the halogen-deuterium light source (Avantes AvaLightD(H)-S), and the second was connected to an AvaSpec-2048 spectrometer. The angular resolution of the setup has a Gaussian shape with half-width of ~ 5° (Stavenga et al. 2020). Illumination angle varied from − 70° to 70° in steps of 10°, recording the reflectance spectra at each illumination position in steps of 10°. A linear polariser mounted on the detection fibre allowed the assessment of the transverse electric (TE) and transverse magnetic (TM) polarisation modes. Owing to the use of a diffuser tile as a reference, the recorded reflectance values frequently exceeded 1. The angle-dependent reflectance spectra across the entire reflection hemisphere for each species were presented as heatmaps, generated by interpolating the mean spectra of measured reflectance for all illumination angles, from − 70° to 70° in 10° steps.

### Imaging scatterometry

The angular distribution of the reflected light was also investigated with an imaging scatterometer (Stavenga et al. 2009; Sosa Espinosa et al. 2024). Wing pieces were glued to the tip of a glass micropipette, oriented perpendicularly to the scale ridges, and positioned at the first focal point of the ellipsoidal mirror of the scatterometer (Supplementary Material, Fig. S0A, B). A xenon light source provided white light illumination. The resulting far-field spatial reflection patterns were recorded with a FLIR BFLY-U3-23S6C-C camera (FLIR Integrated Imaging Solutions, Canada).

## Results

**Fig. 1:**
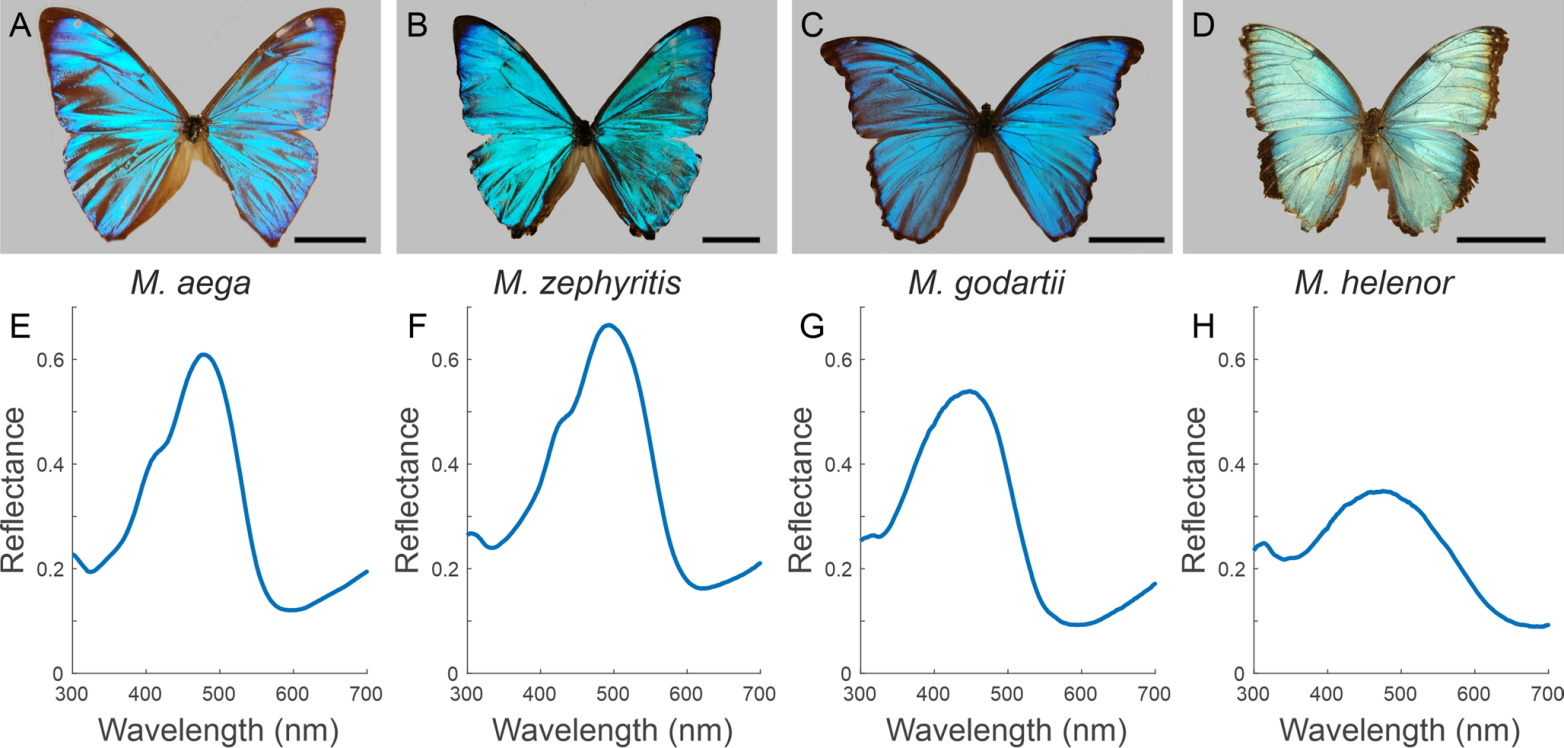
Four similarly coloured *Morpho* butterfly species. **A**–**D** Photographs under normal illumination. Scale bars: **A**,** B** 2 cm; **C**,** D** 4 cm. **E**–**H** Reflectance spectra of the intact wings measured with an integrating sphere. **A**, **E** *M. aega*. **B**, **F** *M. zephyritis*. **C**, **G** *M. godartii*. **D**, **H** *M. helenor*

The wings of the four investigated *Morpho* species, *M. aega*, *M. zephyritis*, *M. godartii* and *M. helenor* exhibit a blue to blue-green colouration (Fig. 1A–D), emerging from the lattice of wing scales. The reflectance spectra recorded from intact wing patches (~ 10 mm diameter) measured with the integrating sphere reveal broadly similar spectral bands within the blue to blue-green range, somewhat varying in amplitude and modulation (Fig. 1E–H).

**Fig. 2:**
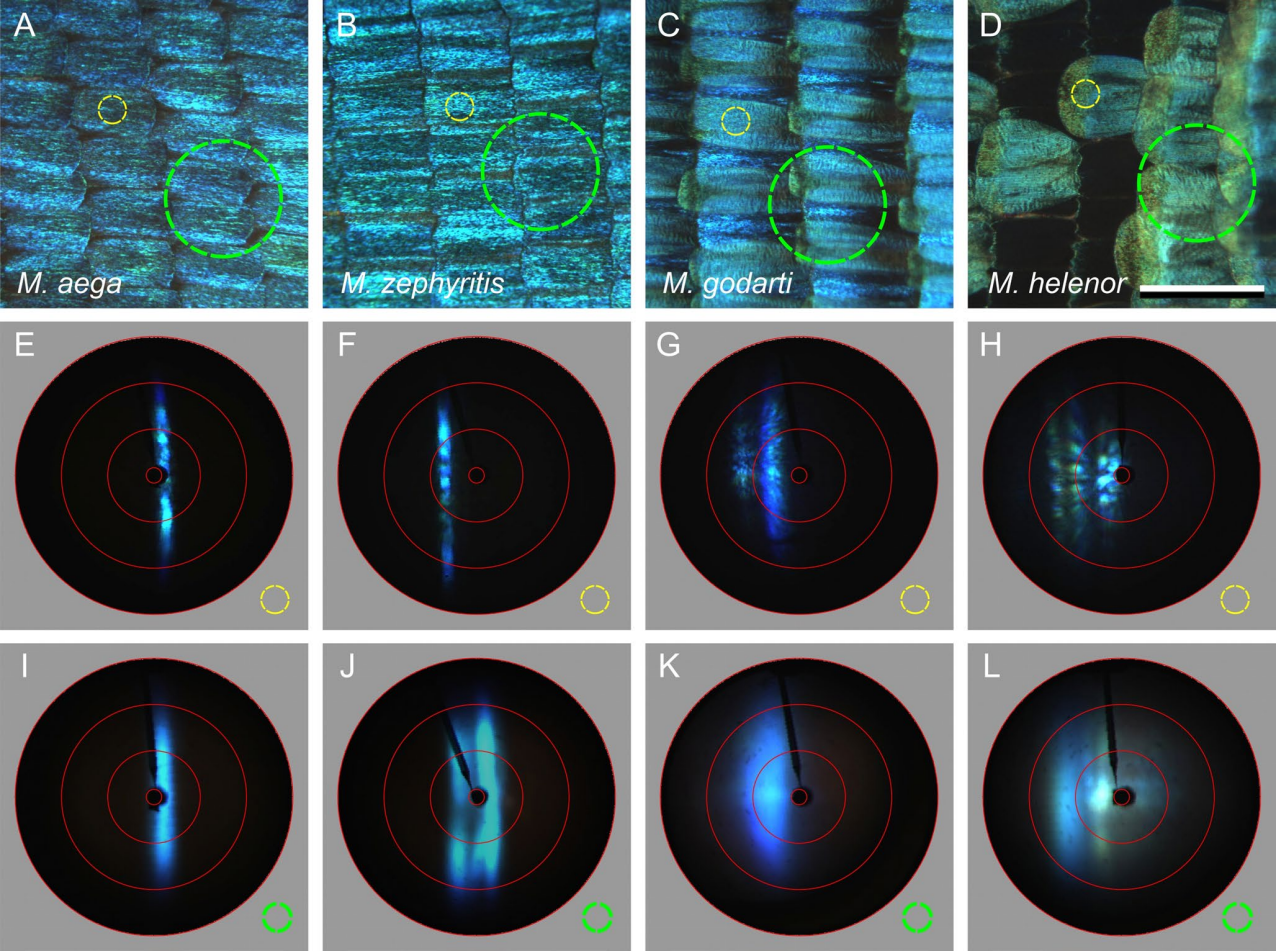
Wing pieces of the four *Morpho* species and scatterograms. **A–D** Small (yellow circles) and large (green circles) wing areas were illuminated. **E–H** Scatterograms of small illumination areas. **I–L** Scatterograms due to large illumination areas. **A**,** E**,** I** *M. aega*. **B**,** F**,** J** *M*. *zephyritis*, the multiple bands observed in **J** are owing to the simultaneous illumination of highly directional cover and ground scales, because of the larger spot size. **C**,** G**,** K** *M. godartii*. **D**,** H**,** L** *M. helenor*; in panel D, dark areas are due to lost cover scales. **A**–**D** Scalebar: 200 μm. **E**–**L** The red circles indicate spatial angles of 5°, 30°, 60°, and 90°

The spatial reflection patterns vary markedly among the studied species, which is likely caused by the variation in the organization of the scales on the wings. We investigated the spatial characteristics by applying imaging scatterometry on dorsal wing pieces that display the dominant iridescent colouration (Fig. 2A–D). Normal illumination of small wing pieces yielded scatterograms with a single blue band for *M. aega* and *M. zephyritis*, but dual and even triple bands emerged for *M. godartii* and *M. helenor* (Fig. 2E–H) (Supplementary Material, Fig. S1). Illuminating a patch of several scales broadened the scatterograms (Fig. 2I–L). The various scatterograms can be well explained from the organization of the scales on the wings (Supplementary Material, Fig. S1). The single layer of flat ground scales in *M. aega* (Fig. 2A) creates a highly directional appearance. By contrast, the stack of half-curved cover scales and a layer of ground scales in the wings of *M. helenor* (Fig. 2D) result in a broader, more spatially uniform reflection pattern in the scatterogram (Giraldo et al. 2016; Sosa Espinosa et al. 2024).

**Fig. 3:**
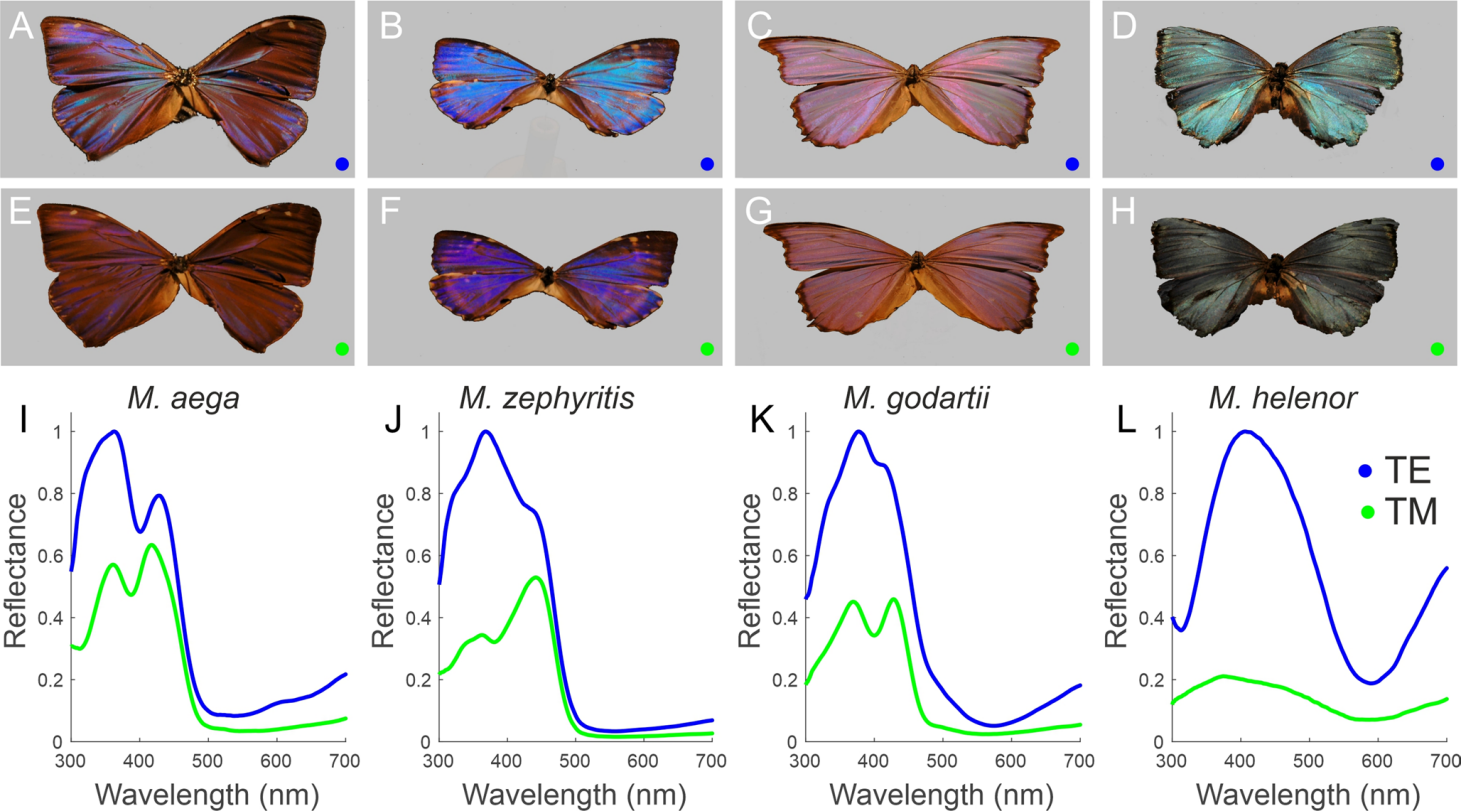
The four *Morpho* species of Fig. 1 illuminated from an angle of ~ 60° and observed at the mirror angle through a polarising filter allowing TE-(**A–D**, blue dot) or TM-(**E–H**, green dot) polarised light. **I–L** Reflectance spectra normalized to the peak reflectance of the TE-spectra (TE; blue curves; TM, green curves) measured when the difference between TE- and TM-reflected light was about maximal, i.e., with illumination angle − 50° for **A**,** E** *M. aega*, **B**,** F** *M. zephyritis* and − 40° for **C**,** G** *M. godartii* and **D**,** H** *M. helenor*

Upon oblique illumination, the wings strikingly varied in brightness and hue and show a polarisation effect (Fig. 3A–H). The angle-dependence of the colouration originates from the multilayered architecture of the wing scales (Zhu et al. 2009; Kambe et al. 2011). The wing reflections become increasingly polarised when increasing the angle of light incidence, with a maximum contrast between TE- and TM-polarised light at Brewster’s angle, i.e. about 60° for chitinous layers in air. To explore the polarisation further, we illuminated the wings of the four species with a narrow-aperture white beam and measured the reflectance spectra for TE- and TM-polarisations (Fig. 3I–L). The reflection angle yielding the highest polarisation contrast varied among species, being about − 50° for *M. aega* and *M. zephyritis*, and about − 40° for *M. godartii* and *M. helenor*. We attribute this variation to the differences in the angular orientation and morphology of the scale lamellae.

Compared to the spectra under normal incidence (Fig. 1E–H), those under oblique illumination exhibited a spectral shift toward shorter wavelengths. In *M. zephyritis*, the spectral shift into the ultraviolet reveals a noticeable lower reflectance in the long-wavelength range compared to the others (Fig. 3J), presumably owing to a higher presence of melanin pigment in the wing scales. As a result, the displayed wing colour is blue to purplish. By contrast, the reflectance of *M. godartii* and *M. helenor* have a higher component in the red wavelength range (Fig. 3K, L), because of the presence of unpigmented cover scales (Fig. 2C, D). The blue colouration of *M. aega* observed in Fig. 1A, E has an extreme shift into the UV under oblique illumination, showing two peaks, with only the lower one visible in the violet/blue range.

**Fig. 4:**
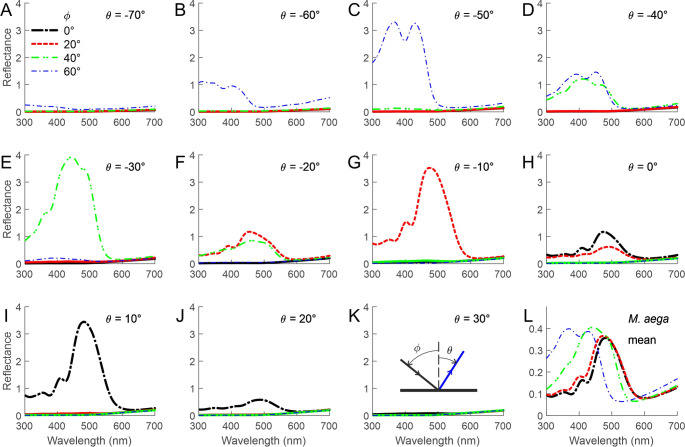
Angle-dependence of the reflectance spectra of a *M. aega* wing. **A**–**K** Reflectance spectra with angle illumination *ϕ* = 0°, 20°, 40° and 60° measured at reflection angles *θ* = -70° to 70° in steps of 10° (inset panel K). Maximum reflectance is reached at 10°, -10°, -30° and − 50°, respectively. **L** Mean of all spectra measured for each of the four illumination angles

The polarisation photographs and spectra suggest that the wing display will strongly vary during flight. This polarised iridescence may provide important clues for intraspecific recognition, especially considering that butterfly spectral sensitivity extends into the ultraviolet (van der Kooi et al. 2021). We therefore further investigated the angle dependence of the wing reflections (Fig. 4). A small wing area of about 3 mm in diameter was illuminated with a narrow-aperture white light beam from various incident angles *ϕ*, with the plane of light incidence parallel to the local scale ridges. We measured the reflectance spectra in the same plane, at reflection angles *θ* varied from − 70° to 70° in steps of 10°.

For *M. aega*, the black curves in Fig. 4 show the spectra obtained for normal illumination, i.e., at *ϕ* = 0°. The reflectance spectrum with maximal amplitude was obtained at reflection angle *θ* = 10° (Fig. 4I), suggesting an effective angular inclination of the scale ridge lamellae close to 5°. At reflection angles *θ* = 0° and 20°, the reflectance amplitudes were much reduced, demonstrating that the reflection is strongly directional, in line with the scatterograms (Fig. 2E, I). Reflections due to illumination at *ϕ* angles of 20°, 40° and 60° showed a similar behaviour, except that the peak reflection is progressively shifting hypsochromically (to shorter wavelengths) (Fig. 4L). In other words, the wing reflection of *M. aega* is highly directional and iridescent. Angle-dependent reflectance measurements on the wings of the other *Morpho* species yielded similar data, with some species-specific nuances (Supplementary Material, Fig. S2-4).

**Fig. 5:**
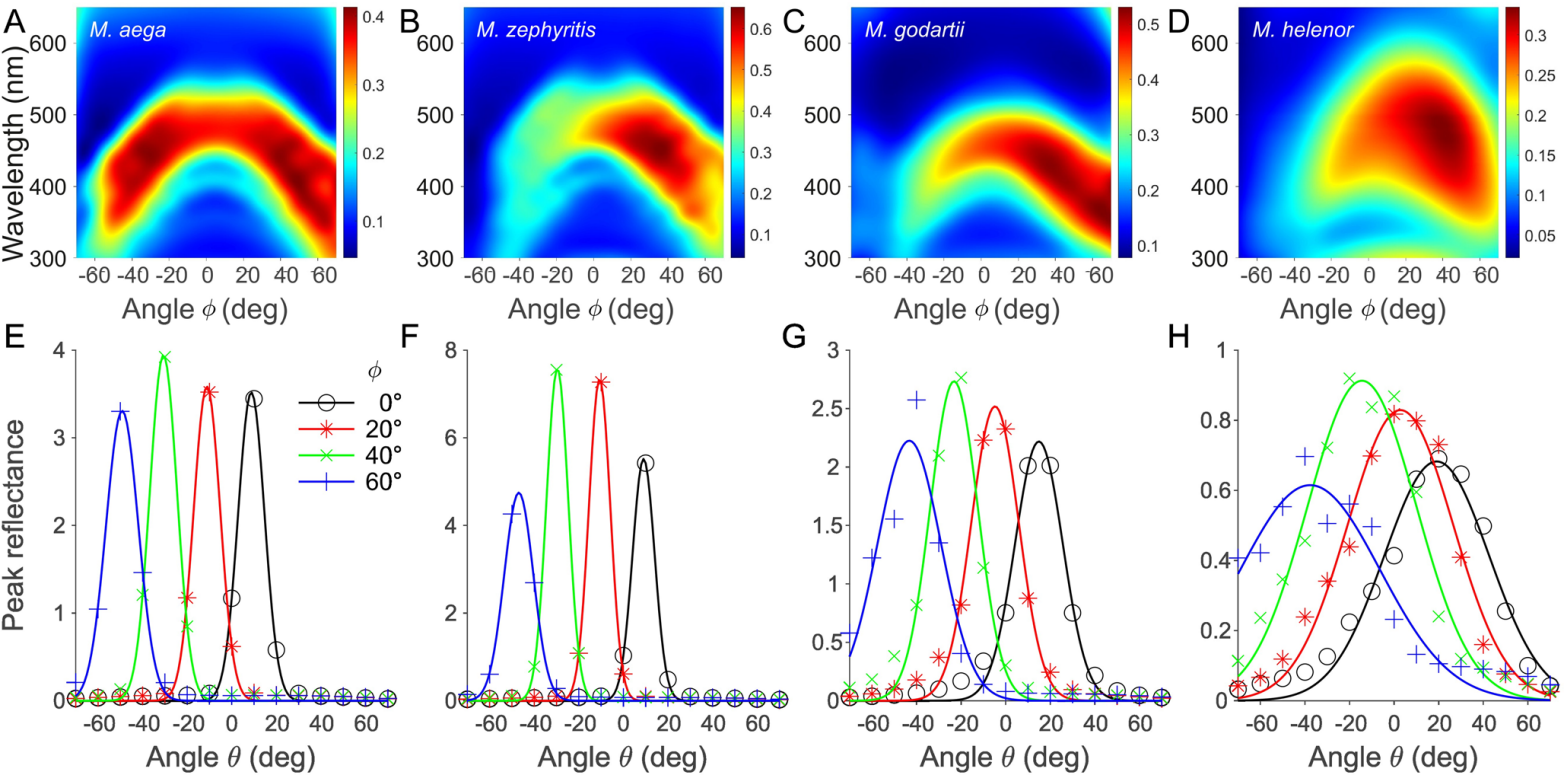
Angle dependence of the wing reflections of the investigated *Morpho* species. **A**–**D** Heatmaps derived from the mean reflectance spectra determined at various illumination angles *ϕ* (as those of Fig. 4L for *M. aega*). **E**–**H** The peak reflectance of the spectra as a function of the reflection angle *θ* measured at illumination angles *ϕ* = 0°, 20°, 40° and 60° (from right to left). Note the differences in y-axis ranges

The angle-dependent reflectance spectra are presented in a concentrated form as heatmaps in Fig. 5. For example, Fig. 5A was composed by interpolating the mean spectra of *M. aega* measured for each illumination angle (from − 70° to 70° in steps of 10°, i.e., as those of Fig. 4L). Figure 5A thus illustrates that for *M. aega*, the peak reflectance shifts about symmetrically towards shorter wavelengths with illumination angles deviating from normal. This is less prominent for the other species, indicating a larger angular spread of the reflected light.

To quantify the angular spread of the reflected light (i.e. the directionality of the visual signal), we assessed the reflectance peak amplitudes measured at the range of reflection angles *θ* for each of the illumination angles *ϕ* = 0°, 20°, 40° and 60° (Fig. 5E–H). The obtained data could be fitted reasonably well with Gaussian functions. Figure 5E shows that for *M. aega* the peak reflectance occurs within a narrow angular range, with a halfwidth of ~ 15°. Comparable angular profiles are shown for *M. zephyritis*, *M. godartii* and *M. helenor*, with halfwidths of ~ 12°, 27°, and 61°, respectively. These values indicate that *M. zephyritis* has the most directional wing reflections, with the lowest halfwidth and highest reflectance (Fig. 5F). By contrast, *M. helenor* exhibits the broadest and least intense reflections (Fig. 5H). All four studied species are iridescent, but the hue shifts are clearly species-specific, due to the quite different angular profiles (Fig. 5E–H). These angular profiles determine whether iridescence appears as flashing or as a more diffuse reflection across the reflection hemisphere, with each possibility potentially serving different ecological roles.

## Discussion

*Morpho* butterfly colouration has fascinated researchers for many decades. Previous work has emphasized the role of scale morphology, the architecture of the wings scales, the arrangements of the multilayered systems and the thicknesses of the reflective surfaces in the final displays (Srinivasarao 1999; Vukusic and Sambles 2003; Yoshioka and Kinoshita 2004; Berthier et al. 2006; Giraldo and Stavenga 2016; Sosa Espinosa et al. 2024).

In the present study, we investigated the directionality (flash effect) and angle-dependence (iridescence) of the reflected light and found distinct species-dependent differences. Directionality refers to the extent to which an objects’ appearance varies with viewing angle, ranging from flashing or diffuse, angle dependence further constrains the viewing geometries that are crucial for an effective display (Stuart-Fox et al. 2021). To understand this variability, we can identify six key optical aspects that contribute to the *Morpho* colour gamut. (i) The wing scale’s lower lamina acts as an optical thin film. The light that is reflected by the lower lamina is typically blue and of low intensity, because the lower lamina consists of a single layer. (ii) Most of the reflected light originates from the multilayer structure via constructive interference (Ghiradella 1984). The “Christmas tree” multilayers typically consist of five to ten superimposed lamellae, which together reflect more light than the lower lamina (Giraldo et al. 2016). Consequently, the multilayers are the main source for the iridescent effect, which is characterised by the shift of the peak reflection to shorter wavelengths with increasing oblique angles of illumination and/or viewing (Figs. 4 and 5). Spatial variation in the multilayer’s dimensions, such as irregular heights of the ridges, will reduce the directionality of the reflected light and cause a more diffuse reflection pattern (Vukusic et al. 1999; Kinoshita et al. 2002; Yoshioka and Kinoshita 2006; Giraldo and Stavenga 2016). (iii) The curvature of the wing scale crucially determines the spatial distribution of the reflected light (Vukusic et al. 1999; Pirih et al. 2011; Sosa Espinosa et al. 2024). Flat scales create bright and directional flashes, whereas curved scales scatter light over a wider angular space. (iv) Wing scale superposition is important for both the intensity of the reflected light as well as its spatial distribution. An aligned stack of flat wing scales will create a bright appearance (Figs. 1 and 2). When the scales are curved, however, superposition of wing scales broadens the angular spread of the display (Vukusic et al. 1999; Pirih et al. 2011; Sosa Espinosa et al. 2024) (Supplementary Material, Fig. S1). Any flashing, iridescent effects that may be observed for individual scales and local illumination will thus be less pronounced in natural conditions. (v) The insertion angle of the wing scale relative to the wing substrate, as well as the effective tilt of the lamellae relative to the lower laminae of the scale, constrain the angle of the reflected light. Although detailed anatomical investigation is required to quantify those angles, our angle-dependent reflectance measurements reveal some species-specific differences. Among the four studied species, the angles under which the polarisation is most noticeable varies with about 10–20°. The tilting of multilayer systems, which maximizes the reflection at specific angles, has been reported in butterflies such as *Ancyluris meliboeus* and *Troides magellanus* (Vukusic et al. 2002; Lawrence et al. 2002). Therefore, the angular reflectance variation within *Morpho* butterflies may also be due to an inclination of the lamellae relative to the lower lamina of the scales (Berthier et al. 2006; Giraldo and Stavenga 2016). (vi) The orientation of the scales on the wing is another critical factor governing the directionality of the display. Consistent with expectations for a predominantly specular response, the highest reflectance is observed when the illumination plane is parallel to the lamellar ridges (Fig. 3). We focused on comparatively small patches of butterfly wings, covering a circular surface area of about 3 mm in diameter, which comprises ~ 200 scales that are aligned in parallel on the butterfly wing patch. However, the orientation of wing scales can vary between parts of the wing, which means that different wing areas reflect light in different directions. This effect is expected to be most important in large butterflies. (vii) Melanin pigmentation enhances the contrast of structural colouration and suppresses incoherent scattering. In iridescent species, melanin may occur in both cover and ground scales, as well as in the wing substrate, like in *M. cypris*. In this species, the white wing stripe results from the absence of melanin in both the scales and the wing substrate (Yoshioka and Kinoshita 2006; Giraldo and Stavenga 2016).

A key question for the evolutionary ecology of *Morpho* and other butterflies is how the optical properties of their wings evolved with regards to the visual systems of their observers and their habitat’s light environment. Natural observers can be potential partners, conspecifics of the same sex (competitors) and predators, such as birds. The visual systems of *Morpho* are only beginning to be understood (Frederiksen and Warrant 2008; Belušič et al. 2021). Existing knowledge on the spectral sensitivity and polarisation vision of other butterflies renders it likely that *Morpho* can perceive the colour and polarisation effects of their wings (Kinoshita et al. 2011; Kinoshita and Arikawa 2014; van der Kooi et al. 2021). In line with this hypothesis, recent behavioural experiments in *M. helenor* suggest that iridescence can provide visual cues used in mate choice (Ledamoisel et al. 2025). Flashing, iridescent displays can constitute conspicuous (long-range) cues because of their abrupt and strong changes in intensity, whereas colour and polarisation cues may be more relevant for short range visual communication (How et al. 2015; Stuart-Fox et al. 2021; van der Kooi and Spaethe 2023; Dietz et al. 2025). Achromatic (intensity-based) visual cues are important for object detection for a suite of insects, including butterflies and moths (van der Kooi and Kelber 2022). The evolution of *Morpho* visual effects might be also shaped by predator-mediated selection. Iridescence and bright flashes, such as those emitted by some *Morpho* species, reduce the accuracy of bird strikes (Pike 2015; Silvasti et al. 2024). Similarly, glossy surface reflections of flowers reduce visibility to insect pollinators (Dietz et al. 2025), and surface gloss hinders tracking of prey-like objects by jumping spiders (Franklin et al. 2024). As there is no compelling evidence that birds perceive polarisation effects, polarised iridescence might constitute a private visual channel, allowing butterflies to exchange visual signals while remaining undetected by predators. Finally, *Morpho* butterflies are found in a wide range of habitats, which may lead to the evolution of divergent visual signalling mechanisms, such as polarised iridescent displays, exploiting the local illumination conditions (Douglas et al. 2007). Therefore, addressing the morphological variations within different species can be a way to further understand how ecology drives the evolution of the ultrastructures that produce such striking iridescent colourations.

The geometry of the illumination (sun), the signalling butterfly and the observer are another crucial aspect to consider (Osorio and Ham 2002; Simpson and McGraw 2018). The display can be markedly different during flapping and gliding flight. This means that flight behaviour, the relative position in the habitat (e.g. understory-flying versus canopy-flying) and time of day are key components to understand the natural dynamics that have shaped the evolution of *Morpho* wing colours (DeVries et al. 2010; Chazot et al. 2021; Le Roy et al. 2021). The evolution of the visual displays of the genus *Morpho* are likely the result of a mix of selective pressures that favour the production of flashing, iridescent signals for intraspecific communication, while producing disruptive signals for (avian) predators (Young 1971, 1973; Vukusic et al. 1999; Pike 2015; Llaurens et al. 2021; van der Kooi and Spaethe 2023). For instance, *M. helenor* exhibits pronounced colour shifts with changes in both illumination and observation angles (perceptible as a broader spread of the heatmap along the y-axis), producing a highly variable optical display spanning ~ 400 to 550 nm across a broad region of the reflection hemisphere (Fig. 5D). This highly dynamic response makes the display well suited for visual signalling. In addition, the signal loss towards negative illumination angles observed for *M. zephyritis*, *M. godartii* and *M. helenor* supports the potential occurrence of an effective multilayer tilting, which skews the display by generating a species-specific horizon of iridescence (Fig. 5A–C) (Vukusic et al. 2002). A comprehensive phylogenetic analysis of the structural and morphological colouration mechanisms will allow a better understanding of the colourful evolution of *Morpho* butterflies.

## Supplementary Information

Below is the link to the electronic supplementary material.

Supplementary Material 1

## Data Availability

Spectrophotometry measurements are available upon request.
