# Supplementary material for: Iridescence, polarisation and directionality of Morpho butterfly displays
Source: J Comp Physiol A Neuroethol Sens Neural Behav Physiol. 2026 Jun 4;212(4):651–60. doi: 10.1007/s00359-026-01817-1 (PMC13396054; doi:10.1007/s00359-026-01817-1)
Supplement: Supplementary file 1 — Supplementary Material 1 [file 359_2026_1817_MOESM1_ESM.docx]

**Journal of Comparative Physiology A (2026)**

**Iridescence, polarisation and directionality of *Morpho* butterfly displays**

Juliana Sosa Espinosa^1,2^, Marco A. Giraldo^2^, Doekele G. Stavenga^1^, Casper J. van der Kooi^1^

^1^Groningen Institute for Evolutionary Life Sciences, University of Groningen, The Netherlands

^2^Biophysics Group, Institute of Physics, University of Antioquia, Medellin, Colombia

Correspondence: [patricia.sosa@udea.edu.co](mailto:patricia.sosa@udea.edu.co)


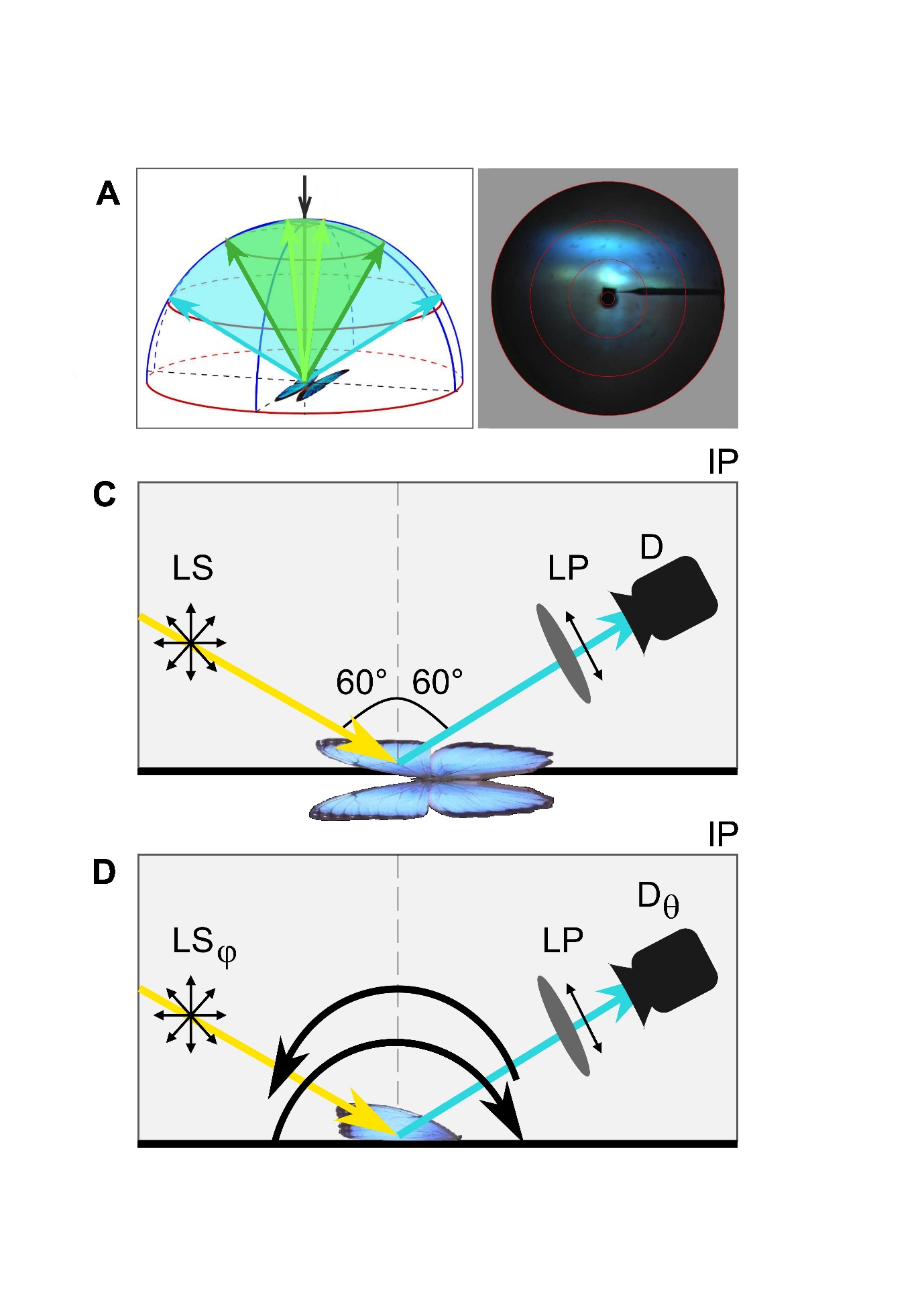


**Fig. S0** Illumination-recapture geometries used in the experiments. **A** Angular spread of reflected light (blue and green arrows) after incident light (black arrow) reaches the sample. **B** Scatterogram of a *M. helenor* wing patch, showing the angular spread of the reflected light as illustrated in A. **C** Illumination geometry of the photography set-up. Both the lightsource (LS) and the detector (D) were fixed at 60°. A linear polariser (LP) was placed before the detector. **D** Illumination geometry of the goniometric set-up. Both the light source and the detection probes scan the illumination plane (IP), with all possible illumination (ϕ) and recapture (θ) angles combinations ranging from -70 to 70 in steps of 10°. A linear polariser (LP) was also placed before the detector.


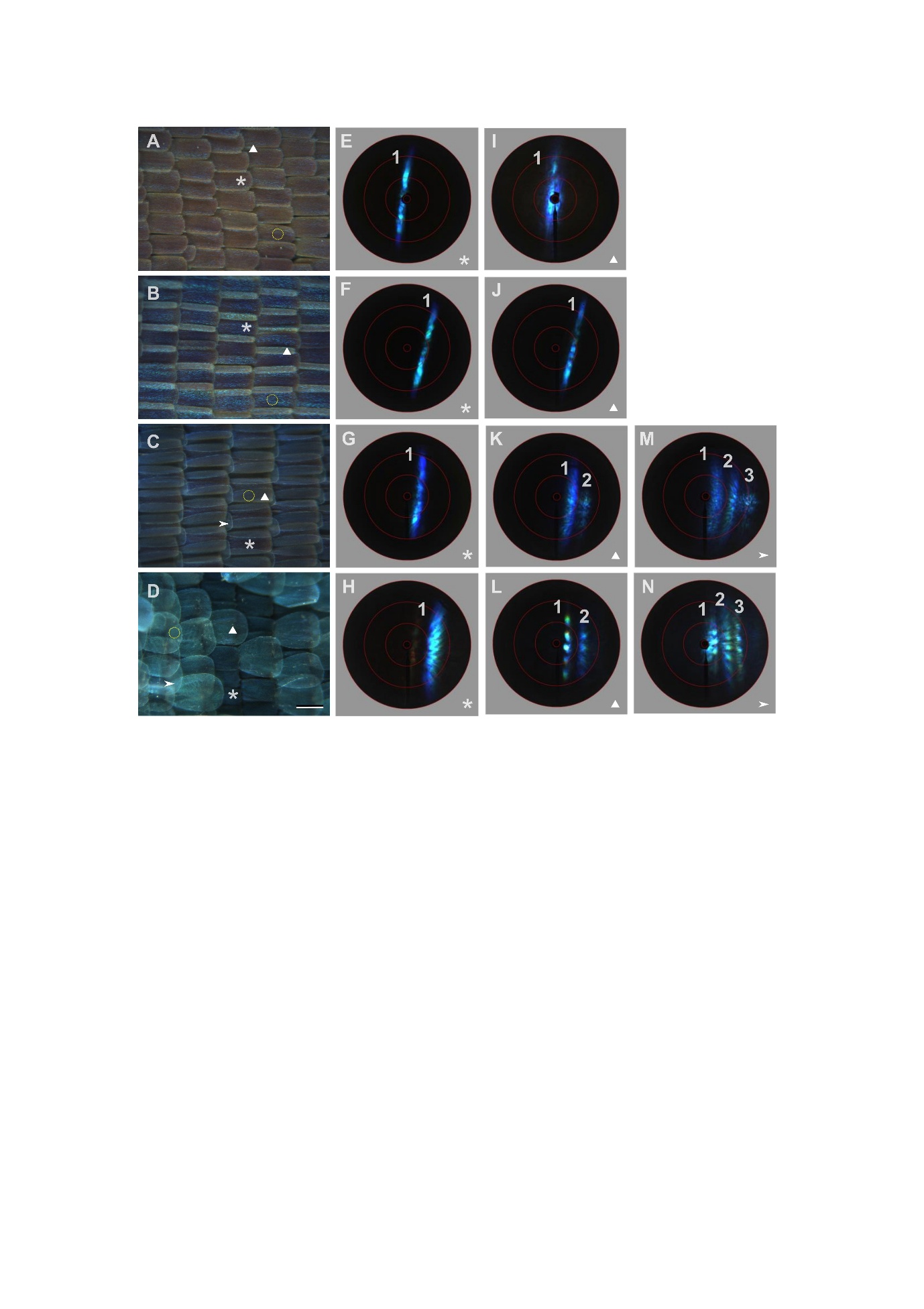


**Fig. S1** Full superposition experiment for the model species. **A-D** Dark field micrographs showing the scale degrees of overlap for **A** *M. aega*, **B** *M. zephyritis*, **C** *M. godartii* and **D** *M. helenor*. Scalebar: 100 µm**.** Asterisk: ground scale, triangle: cover-ground scale overlap; and arrowhead: cover-cover-ground scale overlap (if applicable). **E-N** Narrow aperture scatterograms from alternative scale superposition degrees for **E,I** *M. aega*; **F,J** *M. zephyritis*; **G,K,N** *M. godartii* and **H,L,N** *M. helenor*. The red circles indicate spatial angles of 5°, 30°, 60°, and 90°. Numbers 1, 2 and 3 specify the band numbers for better clarity.


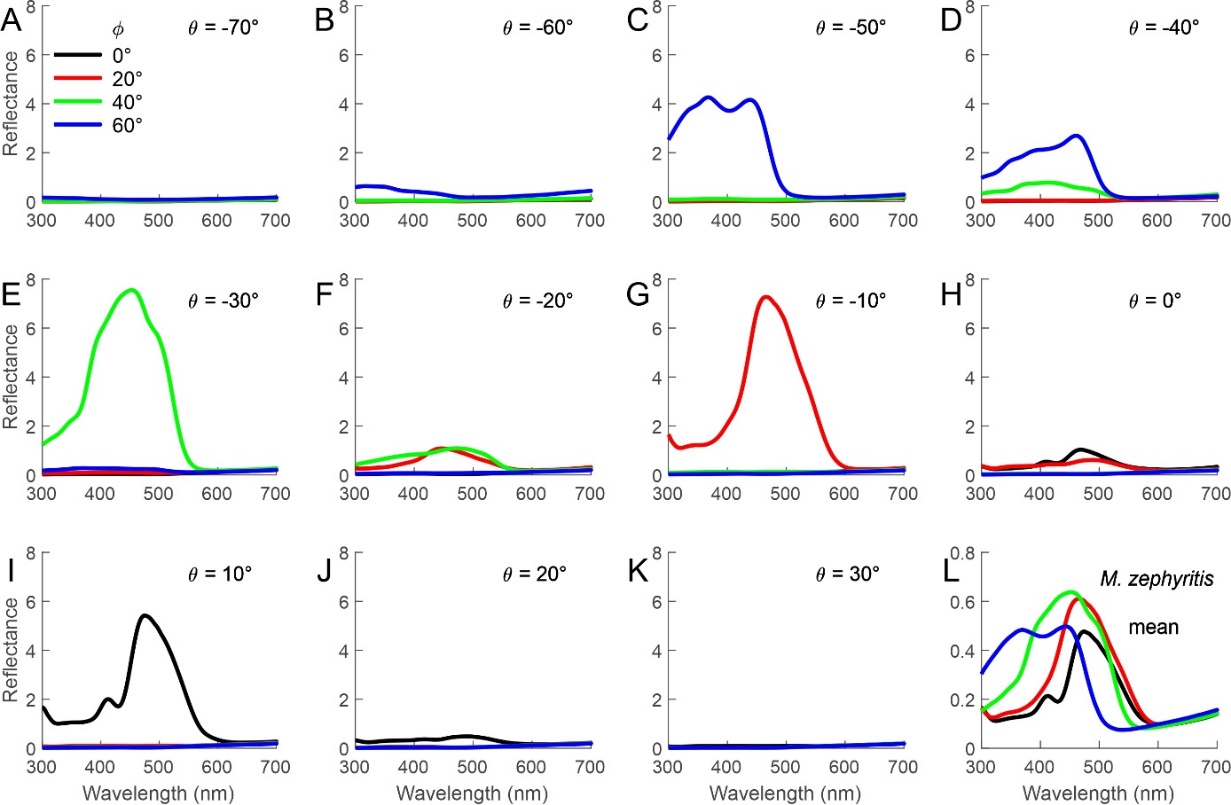


**Fig. S2** Measurements of the angle-dependence of the reflectance spectra of *Morpho zephyritis* wings. Illumination from angular directions 0, 20, 40 and 60° show strongly directional reflections with peak reflection into angles differing 20° from the mirror angle and hypsochromic-shifted spectra with increasing angle of light incidence.


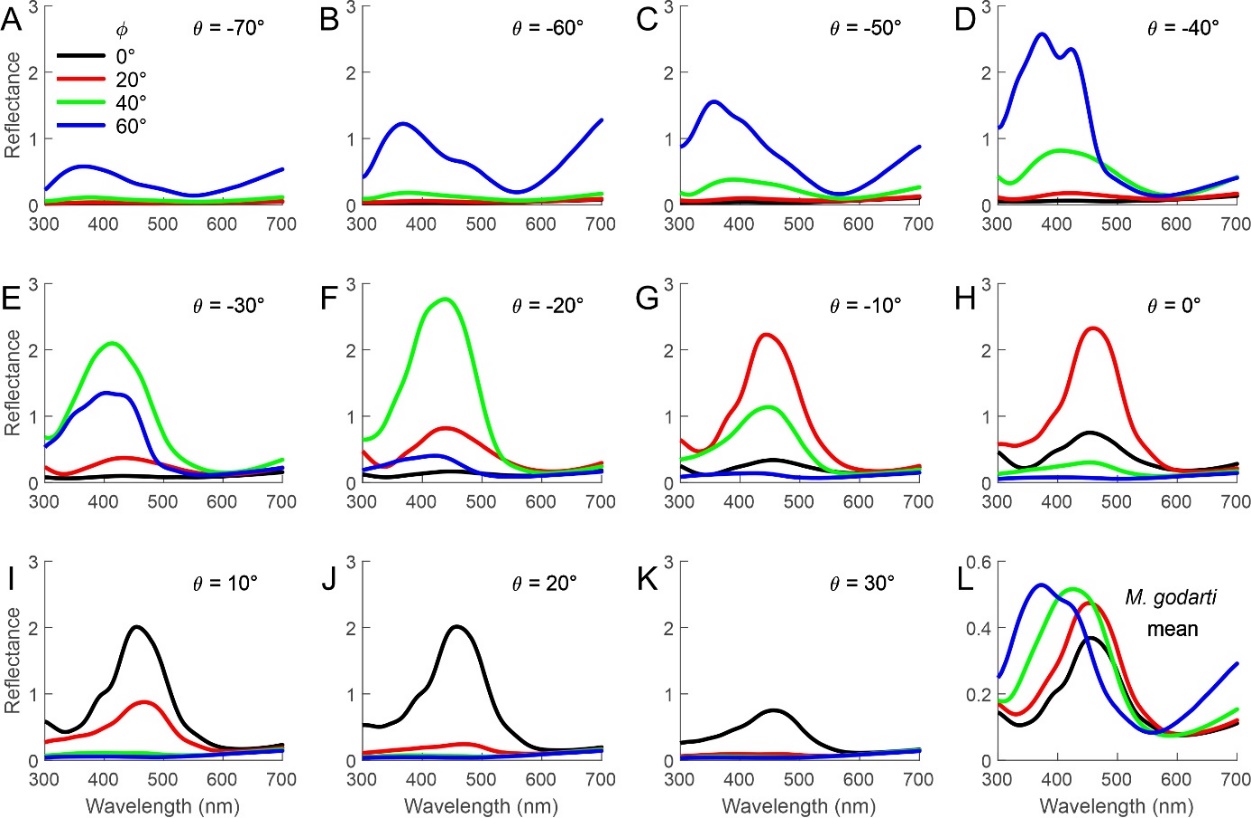


**Fig. S3** As Fig. S2, for *M. godartii*.


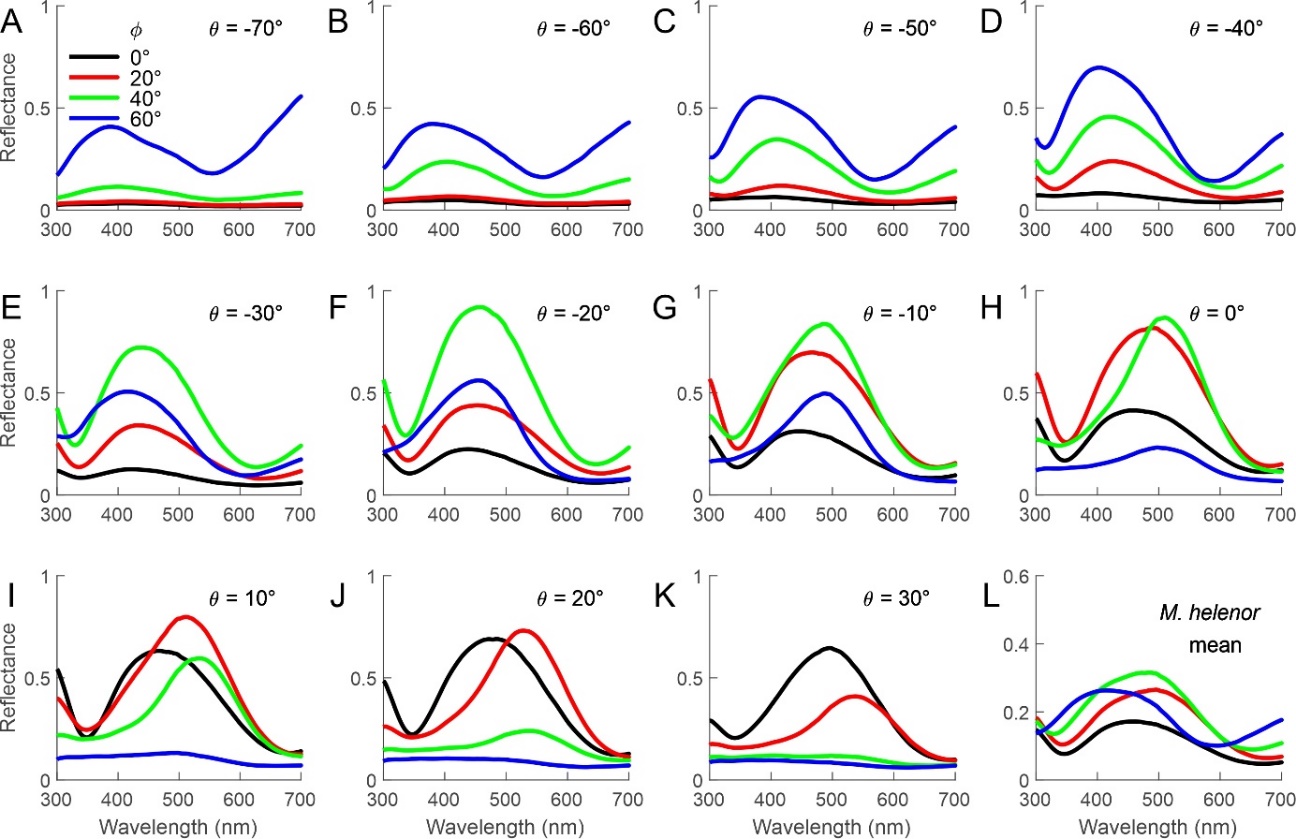


**Fig. S4** As Fig. S2, for *M. helenor*.
